# VCAM-1 and VLA-4 Modulate Dendritic Cell IL-12p40 Production in Experimental Visceral Leishmaniasis

**DOI:** 10.1371/journal.ppat.1000158

**Published:** 2008-09-19

**Authors:** Amanda C. Stanley, Jane E. Dalton, Susanna H. Rossotti, Kelli P. MacDonald, Yonghong Zhou, Fabian Rivera, Wayne A. Schroder, Asher Maroof, Geoff R. Hill, Paul M. Kaye, Christian R. Engwerda

**Affiliations:** 1 Queensland Institute of Medical Research, Herston, Queensland, Australia; 2 Immunology and Infection Unit, Department of Biology, University of York, and The Hull York Medical School, York, United Kingdom; Imperial College London, United Kingdom

## Abstract

Vascular cell adhesion molecule-1 (VCAM-1) interacts with its major ligand very late antigen-4 (VLA-4) to mediate cell adhesion and transendothelial migration of leukocytes. We report an important role for VCAM-1/VLA-4 interactions in the generation of immune responses during experimental visceral leishmaniasis caused by *Leishmania donovani*. Our studies demonstrate that these molecules play no direct role in the recruitment of leukocytes to the infected liver, but instead contribute to IL-12p40-production by splenic CD8^+^ dendritic cells (DC). Blockade of VCAM-1/VLA-4 interactions using whole antibody or anti-VCAM-1 Fab′ fragments reduced IL-12p40 mRNA accumulation by splenic DC 5 hours after *L. donovani* infection. This was associated with reduced anti-parasitic CD4^+^ T cell activation in the spleen and lowered hepatic IFNγ, TNF and nitric oxide production by 14 days post infection. Importantly, these effects were associated with enhanced parasite growth in the liver in studies with either anti-VCAM-1 or anti-VLA-4 antibodies. These data indicate a role for VCAM-1 and VLA-4 in DC activation during infectious disease.

## Author Summary

VCAM-1 and its major ligand VLA-4 are adhesion molecules required for the recruitment and movement of leukocytes within tissue. In this study, we have investigated the role of these molecules during an experimental infection with *Leishmania donovani*, a protozoan parasite that causes a chronic disease called visceral leishmaniasis. Surprisingly, we showed that VCAM-1 and VLA-4 were not required for leukocyte migration into the liver, a site of acute *L. donovani* infection. Instead, there was a requirement for these molecules to initiate cell-mediated immune responses in the spleen within the first 5 hours of infection. When VCAM-1 was blocked during infection, early dendritic cell production of IL-12p40, a potent pro-inflammatory cytokine required for control of *L. donovani*, was suppressed, associated with a reduced parasite-specific T cell response in the spleen, and impaired immunity and parasite clearance in the liver. These results are important because they identify a novel role for VCAM-1 and VLA-4 in the regulation of dendritic cell activation during infectious disease.

## Introduction

Very late antigen-4 (VLA-4; α4 integrin; CD49d) is expressed on most leukocytes and plays an important role in leukocyte trafficking by interacting with vascular cell adhesion molecule-1 (VCAM-1; CD106) on endothelial cells to mediate tethering, rolling, firm adhesion and transendothelial migration [1],[2]. This interaction has also been implicated in the compartmentalisation of B cells into peripheral lymphoid tissue [3], the association of neutrophils with bone marrow (BM) stromal cells [4], the promotion of interactions between follicular dendritic cells (FDC) and B cells [5], and more recently, in the formation of a docking structure that surrounds the B cell receptor (BCR) [6] and TCR [7] in the immunological synapse (IS) that forms between antigen presenting cells and antigen-specific B and T cells.

Visceral leishmaniasis (VL) caused by *Leishmania donovani* infection in genetically susceptible C57BL/6 or BALB/c mice results in parasite replication in the liver, spleen and BM [8]. The liver is the site of an acute, resolving infection, associated with leukocyte recruitment to infected resident Kupffer cells (KC) and the subsequent generation of a localised inflammatory response (granuloma formation), that includes the production of IFNγ, TNF and reactive oxygen and nitrogen species, necessary for killing intracellular parasites [9]–[11]. In contrast, a chronic infection is established in the BM and spleen, associated with major changes to tissue architecture in the latter organ, including the loss of marginal zone (MZ) macrophages and stromal cells in the periarteriolar lymphoid sheath (PALS) [12],[13]. Interestingly, despite long-term parasite persistence in the spleen, this tissue is an important site for early dendritic cell (DC) IL-12p40 production that plays a key role in the generation of anti-parasitic immunity required for the control of hepatic *L. donovani* replication [14]–[16].

We recently showed that VCAM-1 was expressed on hepatic sinusoids during *L. donovani* infection in C57BL/6 mice. Furthermore, we demonstrated that VCAM-1 expression during infection required lymphotoxin alpha (LTα) and that in LTα-deficient C57BL/6 mice, the absence of VCAM-1 was associated with a failure of leukocytes to migrate from periportal areas to infected KC during granuloma formation, coincident with increased parasite growth [17]. In this study, we investigated whether the lack of VCAM-1 expression observed in *L. donovani*-infected mice deficient in LTα could explain their failure to recruit leukocytes into the liver and efficiently control parasite growth. Blockade of VCAM-1 or VLA-4 suppressed anti-parasitic immune responses and was associated with significantly higher hepatic parasite burdens. However, rather than directly mediating cellular recruitment to the liver during VL, our data indicate that VCAM-1 and VLA-4 play a role in rapid CD8^+^ DC IL-12p40 production in the spleen following *L. donovani* infection, an event critical for the generation of effective anti-parasitic immunity.

## Results

### VCAM-1/VLA-4 interactions are critical for efficient control of hepatic *L. donovani* infection

**Figure 1:**
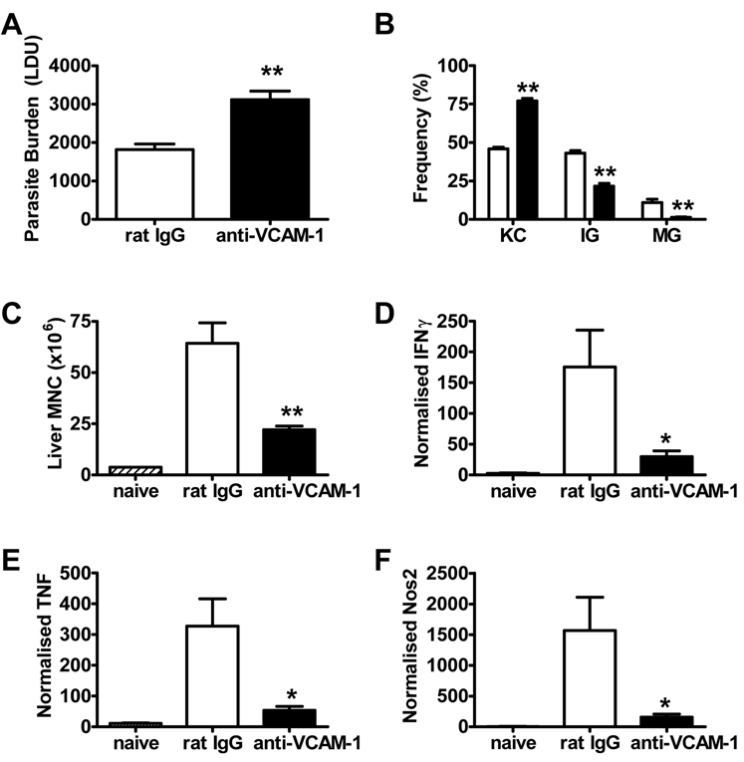
VCAM-1 is required for efficient control of *L. donovani* infection in the liver. Female C57BL/6 mice were treated with anti-VCAM-1 mAb (closed bars) or control rat IgG (open bars) and infected with 2×10^7^ *L. donovani* amastigotes i.v. Mice were injected i.p. with 1 mg of antibody prior to infection and every 3 days thereafter. Parasite burdens were determined in the liver at day 14 p.i. (A) and data represent the mean±SEM in Leishman Donovan units. The number and maturity of hepatic granulomas were estimated on day 14 liver sections stained with anti-*L. donovani* sera (B). Data represent the frequency of infected Kupffer cells (KC), immature granulomas (IG) and mature granulomas (MG) per liver. Hepatic mononuclear cells were isolated from naïve (hatched bars) and infected mice on day 14 p.i. and enumerated (C). mRNA was extracted from livers of naïve or day 14 p.i. VCAM-1 blocked or control mice, and accumulation of IFNγ (D), TNF (E) and NOS-2 (F) mRNA was detected by real-time RT-PCR and is expressed as mRNA molecule per 1000 HPRT molecules. One representative experiment of two performed with similar outcome is shown (n = 4 mice per treatment group in each experiment). Statistical differences of p≤0.05 (*) or p<0.01 (**) for control versus anti-VCAM-1 treatment are indicated.

**Figure 2:**
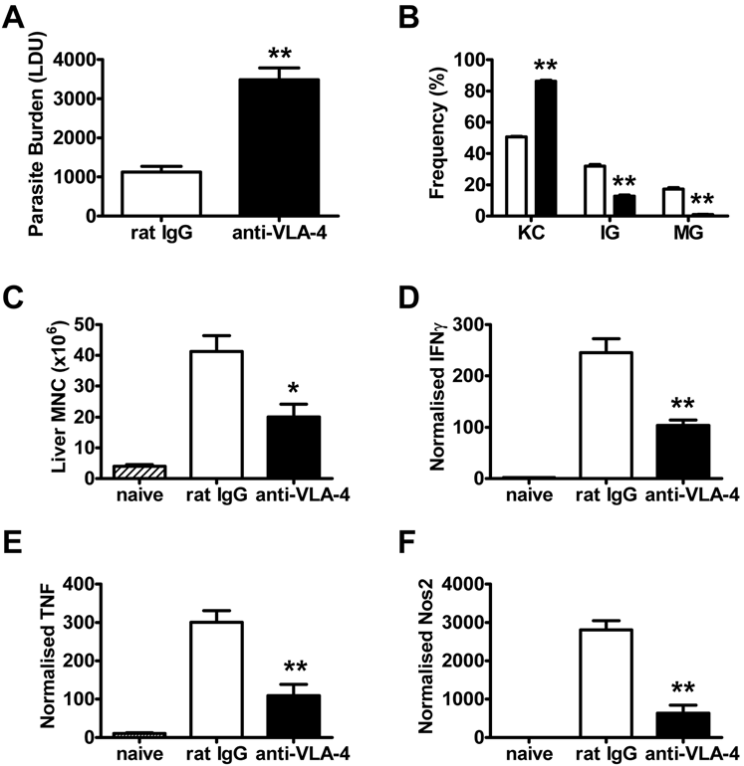
VLA-4 is required for efficient control of *L. donovani* infection in the liver. Female C57BL/6 mice were treated with anti-VLA-4 mAb (closed bars) or control rat IgG (open bars) and infected with 2×10^7^ *L. donovani* amastigotes i.v. Mice were injected i.p. with 1 mg of antibody prior to infection and every 3 days thereafter. Parasite burdens were determined in the liver at day 14 p.i. (A) and data represent the mean±SEM in Leishman Donovan units. The number and maturity of hepatic granulomas was estimated on day 14 liver sections stained with anti-*L. donovani* sera (B). Data represent the frequency of infected Kupffer cells (KC), immature granulomas (IG) and mature granulomas (MG) per liver. Hepatic mononuclear cells were isolated from naïve (hatched bars) and infected mice on day 14 p.i. and enumerated (C). mRNA was extracted from livers of naïve (hatched bars) or day 14 p.i. VLA-4 blocked (closed bars) or control mice (open bars), and accumulation of IFNγ (D), TNF (E) and NOS-2 (F) mRNA was detected by real-time RT-PCR and is expressed as mRNA molecule per 1000 HPRT molecules. One representative experiment of two performed with similar outcome is shown (n = 4 mice per treatment group in each experiment). Statistical differences of p<0.05 (*) or p<0.01 (**) for control versus anti-VLA-4 treatment are indicated.

To investigate the role of VCAM-1 in VL, C57BL/6 mice were administered anti-VCAM-1 mAb 5 hours prior to *L. donovani* infection and then every third day thereafter until 14 days p.i. Hepatic parasite burdens were significantly increased (p<0.01) in these animals compared with rat IgG-treated controls at day 14 p.i. (Figure 1A). In addition, hepatic granuloma formation, an important process for efficient control of *L. donovani* [9], was significantly impaired in these mice with an increase in the frequency of infected KC and a decrease in the frequency of immature (IG) and mature granulomas (MG) (p<0.01; Figure 1B). As expected, this was associated with a dramatic decrease (p<0.01) in leukocyte recruitment to the liver following VCAM-1 blockade (Figure 1C), with the recruitment of all leukocytes studied (CD4^+^ T cells, CD8^+^ T cells, NKT cells, NK cells, B cells, macrophages/monocytes, neutrophils and DC) being significantly and similarly reduced (data not shown). IFNγ, TNF and reactive nitrogen intermediates (RNI; measured using nitric oxide synthase (NOS-2) as a surrogate marker) are all critical for the control of *L. donovani* infection [9]–[11],[17], and mRNA encoding all of these molecules was significantly reduced (p<0.05) in the livers of mice receiving anti-VCAM-1 mAb (Figure 1D–F) at day 14 p.i. To confirm that VLA-4 was the main integrin interacting with VCAM-1 during VL, we also blocked this molecule using antibody over the first 14 days of infection, and obtained very similar results (Figure 2). Together these data indicate that VCAM-1/VLA-4 interactions play an important role in the generation of hepatic immune responses following *L. donovani* infection.

### VCAM-1/VLA-4 interactions do not play a direct role in leukocyte recruitment to the liver following *L. donovani* infection

**Figure 3:**
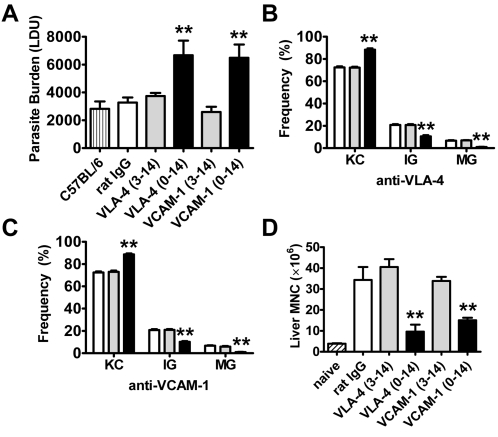
VCAM-1/VLA-4 interactions are not required for leukocyte migration into the liver following *L. donovani* infection. Female C57BL/6 mice were untreated (vertical hatched bar) or treated with anti-VCAM-1 mAb, anti-VLA-4 mAb (closed bars) or control rat IgG (open bars) and infected with 2×10^7^ *L. donovani* amastigotes i.v. Mice were injected i.p. with 1 mg of antibody prior to infection and every 3 days thereafter (black bars), or starting from day 3 and every 3 days thereafter (grey bars). Parasite burdens were determined in the liver at day 14 p.i. (A) and data represent the mean±SEM in Leishman Donovan units. The number and maturity of hepatic granulomas was estimated on day 14 liver sections labelled with anti-*L. donovani* sera from VLA-4 blocked mice (B) and VCAM-1 blocked mice (C). Data represent the frequency of infected Kupffer cells (KC), immature granulomas (IG) and mature granulomas (MG) per mouse. Hepatic MNC were isolated from naïve (hatched bars) and infected mice on day 14 p.i. and enumerated (D). One representative experiment of two performed with similar outcome is shown (n = 4 mice per treatment group in each experiment). Statistical differences of p<0.01 (**) for control versus anti-VCAM-1 or anti-VLA-4 treatment are indicated.

To determine whether VCAM-1/VLA-4 interactions play a direct role in cellular recruitment to the liver following *L. donovani* infection, we delayed VCAM-1 or VLA-4 blockade until 3 days after infection. We chose this time point to begin blockade because no significant cellular recruitment to the liver had occurred before this time (3.85×10^6^±4.48×10^5^ versus 4.22×10^6^±7.86×10^5^ hepatic leukocytes in naïve versus day 3 p.i. mice, respectively). In contrast to blockade commenced prior to infection, this delayed blockade failed to have any effect on parasite burden (Figure 3A), the formation of granulomas (Figure 3B and C), or numbers of hepatic mononuclear cells (MNC; Figure 3D), indicating that VCAM-1/VLA-4 interactions played no role in leukocyte recruitment to the liver. Therefore, the main role for VCAM-1/VLA-4 interactions in VL appeared to be in the generation of immune responses within the first 3 days of *L. donovani* infection.

### Early DC IL-12p40 production in the spleen following *L. donovani* infection

**Figure 4:**
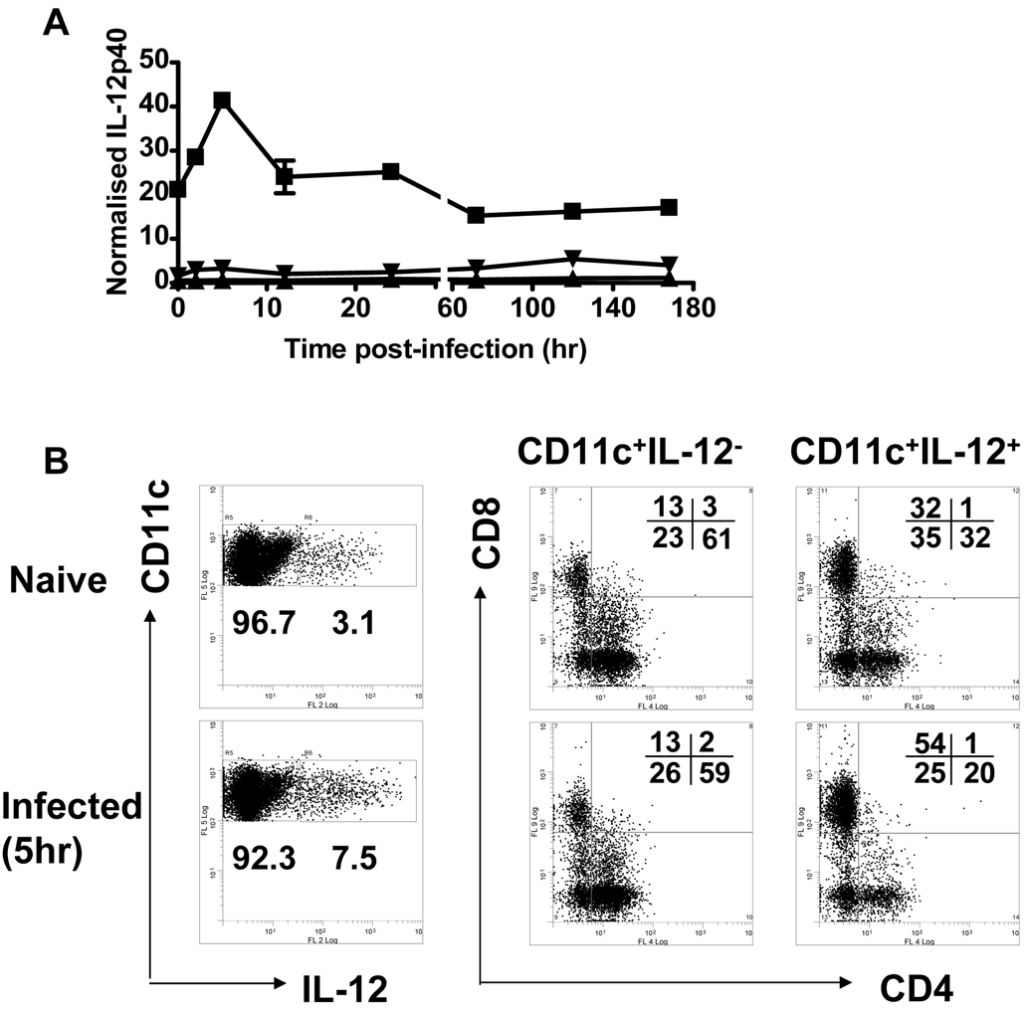
IL-12p40 is produced rapidly and transiently in the spleen by CD8^+^ DC following *L. donovani* infection. (A) Mice were infected with 2×10^7^ *L. donovani* amastigotes i.v. and sacrificed at 2 hr, 5 hr, 12 hr, 24 hr, 72 hr, 5 days and 7 days p.i. mRNA was extracted from the spleen (squares), liver (inverted triangles) and bone marrow (triangles) at each time point and accumulation of IL-12p40 mRNA was detected by real-time RT-PCR and is expressed as mRNA molecule per 1000 HPRT molecules. Data are representative of 3 mice per time point. (B) Mice were infected with 1×10^8^ *L. donovani* amastigotes i.v. and spleens were removed at 5 hr p.i. MACS-enriched DC were enumerated by labelling with anti-CD11c mAb and examined for expression of isotype control mAb or IL-12p40 (1,000,000 events collected, 10,000 events shown). Cells were then electronically gated, based on expression of IL-12p40, as indicated, and then examined for expression of CD4 and CD8 (5000 events shown). Gates were determined from an isotype control for IL-12p40, in which 0.8% of CD11c^+^ cells were shown to be positive. Numbers below rectangular gates indicate the percentage of cells in that gate, while numbers in the upper right hand corners of FACS profiles indicate the percentage of cells in the indicated quadrants. One representative experiment of three performed with similar outcome is shown (n = 4 mice per treatment group in each experiment).

We have previously demonstrated that DC IL-12p40 production in the spleen within 24 hours of *L. donovani* infection is critical for the efficient generation of immunity in the liver [15],[16]. However, the kinetics of DC IL-12p40 production, the relevance of other infected tissue sites for the generation of this cytokine and the specific DC sub-population responsible for IL-12p40 production remain unknown. Therefore, we first measured IL-12p40 mRNA levels in the spleen, liver and BM, the main sites for *L. donovani* infection, over the first 7 days of infection. We found that IL-12p40 production occurred predominantly in the spleen and peaked at 5 hours p.i. (Figure 4A). This pattern of production was also confirmed by analysis of IL-12p40 protein expression in spleen tissue sections (data not shown), as previously described [15]. The IL-12p40-producing DC were located in the PALS, as well in close proximity to the MZ (data not shown), as previously reported [15],[18]. To determine the specific DC subset producing IL-12p40, MACS-enriched DC from naive mice and *L. donovani*-infected mice 5 hours p.i., were labelled for CD11c, CD4 and CD8α, and intracellular IL-12p40 protein levels were measured by FACS. No IL-12p40 protein was detected in CD11c-negative cells from either naïve or infected animals (data not shown), thereby identifying DC as the main source of IL-12p40. Small numbers of IL-12p40-producing CD11c^+^ DC were observed in naïve mice (Figure 4B). Five hours after *L. donovani* infection, there was a 2–3 fold increase in the number of IL-12p40-producing CD11c^+^ DC, and virtually all of the increased IL-12p40 production could be attributed to the CD8^+^ DC subset (Figure 4B). Together, these data demonstrate that parasite-induced IL-12p40 is produced in the spleen by CD8^+^ DC within 5 hours of *L. donovani* infection.

### VCAM-1 co-localises with sinusoidal endothelial cells and MZ macrophages in close proximity with DC in the MZ of the spleen

**Figure 5:**
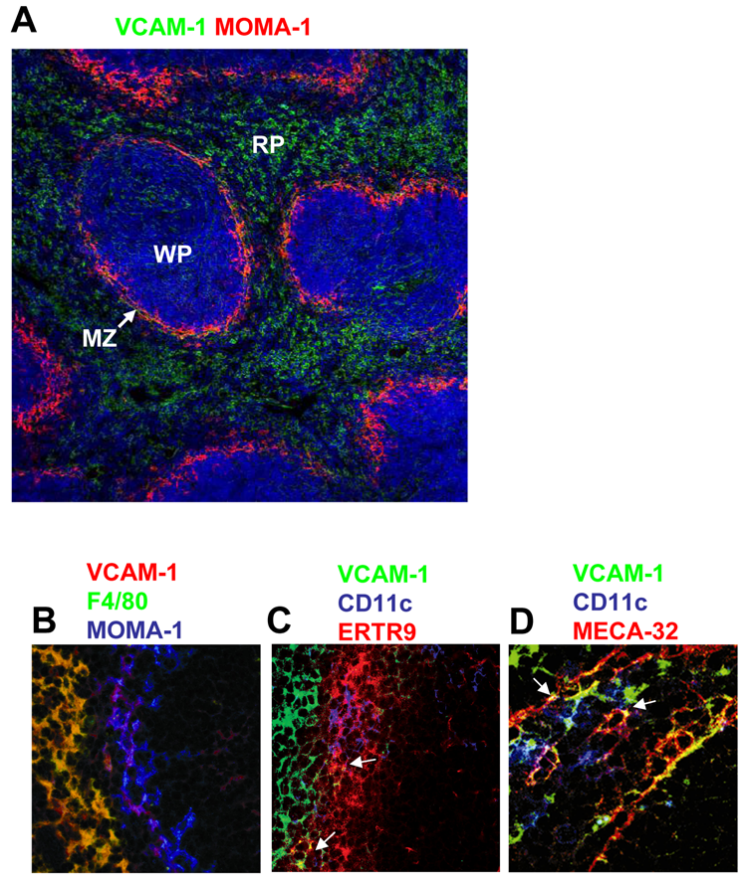
VCAM-1 expression in the spleens of naive C57BL/6 mice. (A) VCAM-1 localisation (green) in a tissue section from a naïve mouse. Marginal metallophilic macrophages were stained with MOMA-1 mAb (red) allowing visualisation of red pulp (RP), white pulp (WP) and marginal zone (MZ) regions of the spleen, as indicated (×100) The tissue section was mounted in media containing DAPI to stain cell nuclei (blue). (B-D) Staining for VCAM-1 expression, red pulp macrophages (F4/80^+^), MM macrophages (MOMA-1^+^), MZ macrophages (ERTR9/SIGNR1^+^), MZ sinus-lining endothelial cells (Meca-32^+^) and DC (CD11c^+^) (×630) is shown in colours indicated above panels. White arrows in (C) and (D) indicate areas where VCAM-1^+^ cells (yellow) are in close proximity to DC (blue).

We next investigated where VCAM-1 was expressed in the spleen to identify cell populations that might influence the generation of immune responses to *L. donovani* infection, and in particular CD8^+^ DC IL-12p40 production. Studies in the mouse spleen have reported VCAM-1 expression associated with individual cells in the red pulp [19], within a broad zone in the MZ [19] and within the B and T cell zones, including on follicular dendritic cells [20]. We confirmed that the majority of VCAM-1 expression was in the red pulp region, and demonstrated that expression could also be detected on discreet cell populations in the MZ and white pulp (Figure 5A). In the red pulp, all F4/80^+^ macrophages expressed VCAM-1 (Figure 5B). However, blood flowing into the spleen is released into the marginal sinus before flowing across the MZ into the red pulp and returning to the circulation via a venous route [21], and the site where DC are first likely to encounter parasites and/or parasite antigens is the MZ [15],[18]. In the MZ, there was little overlap observed between staining for VCAM-1 and marginal metallophilic macrophages (MOMA-1^+^) (Figure 5B). In contrast, there was clear co-localisation of VCAM-1 expression with MZ macrophages (ERTR9^+^/SIGNR1^+^) (Figure 5C), and with sinusoidal endothelial cells (Meca-32^+^) located in the MZ (Figure 5D). Both VCAM-1^+^ MZ macrophages and endothelial cells were located in close proximity to discrete areas of DC (CD11c^+^) (Figures 5C and D). However, no clear VCAM-1 expression was observed on these DC (Figures 5C and D), a finding also confirmed by FACS analysis of VCAM-1 expression by splenic DC (data not shown). There was some co-localisation of VCAM-1 with reticular fibroblasts (ER TR7^+^) throughout the spleen (data not shown). Isotype control mAbs matched in concentration to each mAb did not show any staining (not shown). These results indicate that the major VCAM-1^+^ cells found in the MZ where DC expressing VLA-4 are first likely to contact parasites and/or parasite antigens are the sinusoidal endothelial cells and MZ macrophages.

### VCAM-1/VLA-4 interactions are not required for lymphocyte or DC migration into the spleen

**Figure 6:**
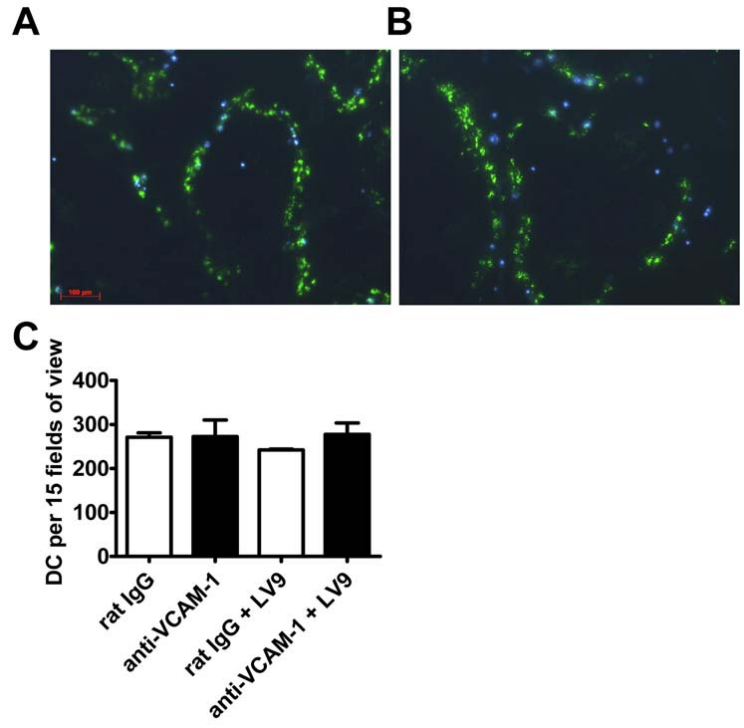
VCAM-1/VLA-4 interactions are not critical for DC migration into the spleen. Naïve C57BL/6 mice were injected with 100 µg FITC-dextran i.v. followed by 1 mg of control rat IgG (A) or anti-VCAM-1 mAb (B) i.p. 24 hr later. Hoechst 33342-labelled splenic CD11c^+^ DC (1×10^6^) were administered i.v. 1 hr following mAb injection. The following day mice were either left as naïve or infected with 2×10^7^ *L. donovani* amastigotes i.v. and spleens were removed 5 hr later (24 hr post-cell transfer). The distribution of Hoechst 33342-labelled cells was analysed in 20 µm sections and photographed under UV illumination (×100). Data are representative of one of two experiments performed (n = 3 mice per group). The number of Hoechst 33342-labelled cells was determined from 15 fields of view per mouse spleen (×25 magnification) (C).

We next investigated whether VCAM-1 blockade modulated DC and/or lymphocyte trafficking in the spleen following *L. donovani* infection. Previous work showed that lymphocyte entry into the spleen was not prevented by integrin blocking antibodies [3], and we also confirmed this result by measuring migration of labelled lymphocytes into the PALS following VCAM-1 blockade in naïve animals and after 5 hours of *L. donovani* infection (data not shown). However, the role of cell adhesion molecules in DC entry into the spleen has not been previously investigated. Therefore, we monitored migration of labelled DC into the spleen following VCAM-1 blockade in naïve mice and after 5 hours of *L. donovani* infection, but found no role for VCAM-1 in this process, regardless whether mice were infected or not (Figure 6A–C). Therefore, VCAM-1 blockade did not affect lymphocyte or DC migration into the spleen, although our data do not exclude a role for VCAM-1 in cell movement between distinct regions within the spleen.

### VCAM-1/VLA-4 interactions play a critical role in splenic DC IL-12p40 production

**Figure 7:**
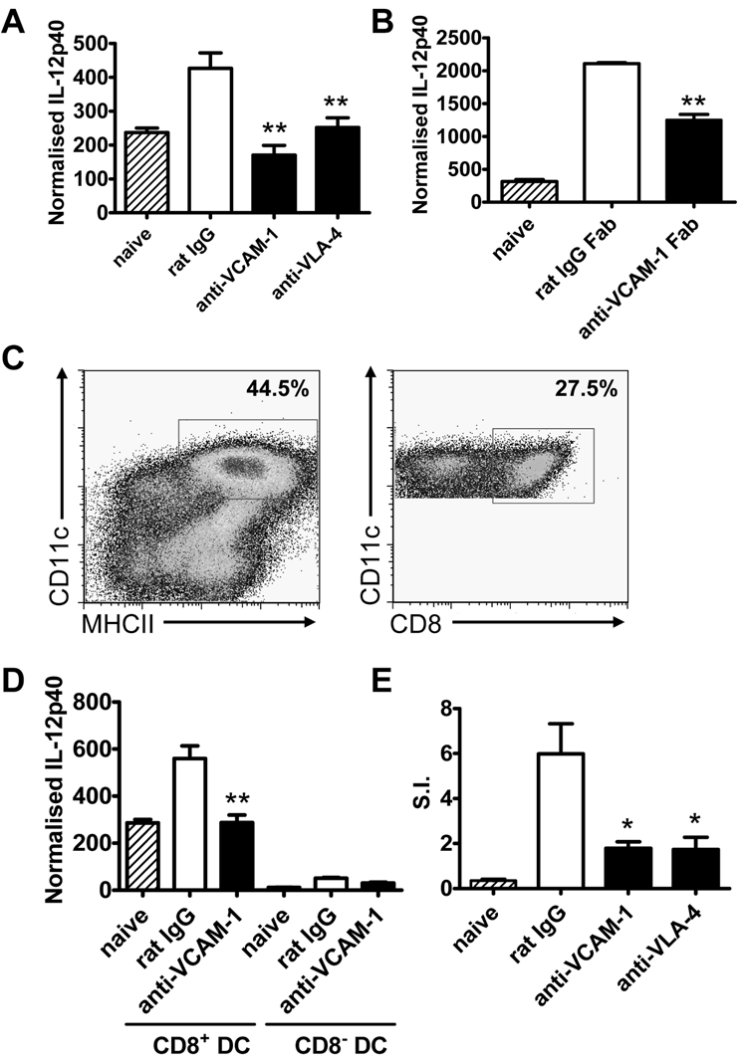
VCAM-1/VLA-4 interactions are required for parasite-induced IL-12p40 mRNA accumulation by CD8^+^ DC. (A) C57BL/6 mice were treated with 1 mg anti-VCAM-1 mAb, anti-VLA-4 mAb or control rat IgG or (B) with 1 mg anti-VCAM-1 Fab fragments or control rat IgG Fab fragments the day prior to infection with 1×10^8^ *L. donovani* amastigotes. CD11c^+^ DC were enriched by positive selection by MACS from the spleens of naïve mice (hatched bars), or antibody treated mice (closed bars) or control treated mice (open bars) at 5 hr p.i. mRNA was extracted from MACS-enriched DC, and accumulation of IL-12p40 mRNA was detected by real-time RT-PCR. One representative experiment of four performed with similar outcome is shown (n = 4 mice per treatment group in each experiment). All data is expressed as IL-12p40 mRNA molecules per 1000 HPRT molecules. (C–D) MACS-enriched CD11c^+^ DC from naïve C57BL/6 mice (hatched bars) or *L. donovani*-infected mice at 5 hr p.i. treated with either control rat IgG (open bars) or anti-VCAM-1 (closed bars), were sorted based on CD11c and MHC-II expression, followed by separation into CD8α-positive and CD8α-negative populations. Percentages of each gated population are indicated. (D) mRNA was extracted from purified CD8^+^ or CD8^−^ DC populations, as indicated, and accumulation of IL-12p40 mRNA was detected by real-time RT-PCR. Data represent groups of pooled cells from 4 mice, repeated 3 times. (E) Proliferation of splenic CD4^+^ T cells from naïve (hatched bars) or *L. donovani*-infected C57BL/6 mice (day 14 p.i.) that had received anti-VCAM-1 mAb, anti-VLA-4 mAb (closed bars) or control rat IgG (open bars), as indicated, in the presence of naïve irradiated splenic APC pulsed with fixed *L. donovani* amastigotes. One representative experiment of two performed with similar outcome is shown (n = 4 mice per treatment group in each experiment). Data is presented as a stimulation index (SI) calculated by dividing the proliferation of each sample in response to parasite antigen by proliferation in culture media alone. Statistical differences of p<0.01 (**) for antibody versus control treated mice are indicated.

To test whether VCAM-1/VLA-4 interactions play a direct role in DC IL-12p40 production, we blocked these molecules 12 hours prior to infection and measured IL-12p40 mRNA levels in DC isolated from the spleen at 5 hours p.i. (Figure 7A). In control-treated mice, DC IL-12p40 mRNA levels increased 2–3 fold 5 hours after *L. donovani* infection. VCAM-1 blockade inhibited 50–100% of DC IL-12p40 mRNA accumulation, while VLA-4 blockade reduced DC IL-12p40 mRNA levels by 50–90% (n = 4 experiments).

However, given the co-localisation of DC with VCAM-1^+^ cells in the spleen (Figure 5), it is possible that the ligation of Fcγ receptors (FcγR) on DC by anti-VCAM-1 mAbs could suppress IL-12p40 production, as previously reported for human and mouse DC [22],[23]. Therefore, we next generated anti-VCAM-1 Fab′ fragments that comprised the antigen binding region of the mAb, but have all FcγR binding domains removed, and hence, are unable to signal via FcγR. Splenic DC isolated from mice treated with control rat IgG Fab′ fragments 5 hours after *L. donovani* infection had a significant accumulation of IL-12p40 mRNA, relative to DC from naïve mice (Figure 7B). Importantly, IL-12p40 accumulation in splenic DC from mice treated with anti-VCAM-1 Fab′ fragments was significantly reduced (p<0.01), compared with DC from control-treated mice (Figure 7B). The reduction of IL-12p40 mRNA accumulation with anti-VCAM-1 Fab′ fragments was not as effective as with anti-VCAM-1 mAb, and this most likely reflects the very short half-life of Fab′ fragments in plasma (around 1 hour) compared with mAbs (hours-days), and their rapid excretion by the kidney [24],[25]. Nevertheless, these data support the conclusion that specific blockade of VCAM-1 reduces DC IL-12p40 mRNA accumulation following *L. donovani* infection, and that this effect of anti-VCAM-1 mAb treatment was not caused by FcγR ligation on DC.

To confirm that parasite-induced IL-12p40 mRNA accumulation in CD8^+^ DC was the main target of VCAM-1 blockade, we next sorted these cells following MACS enrichment, based on expression of CD11c, MHC-II and CD8α (Figure 7C), from naïve animals or at 5 hours p.i. from mice infected with *L. donovani* that had received control rat IgG or anti-VCAM-1 mAb prior to infection. As expected, the majority of IL-12p40 mRNA accumulation occurred in CD8^+^ DC, and levels increased approximately 2-fold at 5 hours p.i. Importantly, this increase did not occur in CD8^+^ DC from mice in which VCAM-1 had been blocked (Figure 7D). There was some disparity between IL-12p40 mRNA accumulation (Figure 7D) and IL-12p40 protein levels (Figure 4B) in different DC subsets from naïve mice, whereby virtually all IL-12p40 mRNA accumulated in CD8^+^ DC, but IL-12p40 protein expression was similar between DC subsets. This may reflect different rates of IL-12p40 mRNA turnover or protein retention in different splenic DC subsets. We also observed that a small proportion of CD8^+^ DC were infected with *L. donovani* amastigotes at 5 hours p.i., and that infection of these cells was not reduced by anti-VCAM-1 mAb (0.50±0.10% versus 0.37±0.11% for mice receiving rat IgG or anti-VCAM-1 mAb, respectively, determined from cytospins).

Our data indicate that VCAM-1/VLA-4 interactions are important for splenic CD8^+^ DC IL-12p40 production 5 hours after *L. donovani* infection, and that this is important for the efficient generation of anti-parasitic T cell responses required for control of hepatic infection. To directly examine whether *L. donovani*-specific CD4^+^ T cell activation was affected by blockade of VCAM-1/VLA-4 interactions, we next isolated splenic CD4^+^ T cells at day 14 p.i., and re-stimulated them *in vitro* in the presence of naïve, irradiated APC and fixed *L. donovani* amastigotes. There was little proliferation of splenic CD4^+^ T cells from naïve animals in response to parasite antigen (Figure 7E). However, significant parasite-specific proliferation was observed in CD4^+^ T cells from control-treated mice, but this was significantly reduced in CD4^+^ T cell from animals that had received either anti-VCAM-1 or anti-VLA-4 mAbs (Figure 7E). There was no difference in CD4^+^ T cell proliferation in response to concanavalin A in any of the groups tested, indicating that blockade of VCAM-1/VLA-4 did not generally suppress or inactivate CD4^+^ T cells. Together, these data indicate that CD8^+^ DC require VCAM-1/VLA-4 interactions for IL-12p40 production, associated with the generation of effective anti-parasitic CD4^+^ T cell responses required for the control of *L. donovani* growth in the liver.

## Discussion

The interaction between VCAM-1 and VLA-4 appears to be important for the outcome of hepatic *L. donovani* infection. However, these molecules play no role in the recruitment of leukocytes to the infected liver during VL, and there appears to be no clear role for LTα-dependent VCAM-1 expression on hepatic sinusoids. Instead, VCAM-1/VLA-4 interactions modulate IL-12p40 production by CD8^+^ DC in the spleen within hours of parasite challenge. Blockade of VCAM-1, along with its physiological ligand VLA-4, resulted in reduced IL-12p40 production by CD8^+^ DC. Studies with anti-VCAM-1 Fab′ fragments also indicate a role for VCAM-1/VLA-4 interactions in DC IL-12p40 production. Blockade of VCAM-1 with antibodies was also associated with reduced CD4^+^ T cell proliferation in the spleen and impaired resistance to *L. donovani* in the liver. The spleen harbours a relatively low parasite burden at the early time points assessed in this study, and this was not affected by VCAM-1 or VLA-4 blockade (data not shown).

DC IL-12p40 production shortly after *L. donovani* infection plays a key role in the generation of anti-parasitic immune mechanisms and is critical for the effective control of VL [14],[16]. Recently, both IL-12p70 and IL-23, each utilising the IL-12p40 subunit, were found to be functionally important cytokines for the control of *L. donovani* infection [26]. We have demonstrated that splenic CD8^+^ DC are the major source of IL-12p40 following *L. donovani* infection, and that parasite-induced IL-12p40 production occurs transiently, peaking 5 hours after infection (Figure 4). This early IL-12p40 production is physiologically important because when it is blocked during the first 24 hours of infection there is a failure to effectively control parasite growth in the liver and spleen [16].

The location of DC in the spleen is important during this early phase of infection and DC are required to migrate from the MZ into the PALS within the first 5 hours of infection in order for efficient T cell priming and maximal IL-12p40 production to occur [18]. This pattern of cell movement supports a model whereby parasites are rapidly taken up by macrophages in the MZ [15],[18], parasite antigen is then either transferred from these cells to DC or the DC directly acquire antigen in the MZ, and subsequently migrate into the PALS for T cell activation. Our data suggest that VLA-4/VCAM-1 interactions play a role in these events. Blockade of VCAM-1 did not affect splenic parasite burden in the first 24 hours of *L. donovani* infection (data not shown), suggesting that VLA-4/VCAM-1 interactions play no role in parasite uptake by macrophages. In addition, the acquisition of parasites by CD8^+^ DC was not prevented by VCAM-1 blockade. Histological examination of spleen tissue indicated that red pulp macrophages were the main VCAM-1^+^ cell population in the spleen (Figures 5A and B). However, these cells are spatially segregated from the IL-12p40-producing DC found in the MZ and T cell zones. In the MZ, sinus lining endothelial cells and MZ macrophages express VCAM-1, and importantly, are found in close proximity to DC (Figures 5C and D). VCAM-1 on endothelium can mediate cell adhesion and transendothelial migration [1],[2], and early in *L. donovani* infection endothelial VCAM-1 may be involved in either the adhesion of DC in the MZ or the movement of these cells into the PALS after antigen acquisition. However, previous work has shown that lymphocyte entry into the spleen is not prevented by blocking any single integrin, including VLA-4 [3], and we also observed no effect of VCAM-1 blockade on lymphocyte trafficking (data not shown) or on the retention of labelled naïve DC in the spleen in naïve animals and 5 hours after *L. donovani* infection (Figure 6). Therefore, a role for VCAM-1-mediated naïve DC retention in the MZ is unlikely.

The other main VCAM-1^+^ cell population in the MZ were the MZ macrophages (Figure 5C). These cells are highly phagocytic and rapidly take up *L. donovani* after infection [15],[18]. Furthermore, these cells are lost from the spleen after a chronic infection becomes established (day 21–28 p.i.) via a TNF-dependent mechanism, disrupting cellular movement in this organ [12]. Therefore, VCAM-1 on MZ macrophages could mediate DC movement from the MZ into the PALS. Alternatively, it could mediate interactions between DC and MZ macrophages, allowing uptake of parasite antigen by DC and/or activation of DC. Attempts to adoptively transfer splenic DC (5×10^6^) isolated from mice 5 hours post-*L. donovani* infection (time of peak IL-12p40 production) to mice receiving VCAM-1 blockade, thereby bypassing these early cellular interactions, failed to improve control of parasite growth, relative to control animals (data not shown). Although this result suggests that later cellular interactions might be VCAM-1 dependent, only a small proportion of transferred DC (less than 10%) were found in the spleen 24 hours after transfer. Therefore, we cannot exclude the possibility that the failure to overcome VCAM-1 blockade resulted from altered DC trafficking caused by infection or the fact that DC from infected mice are unable to traffic effectively back to the T cell zones within the spleen.

VLA-4 has also been localised at the centre of the peripheral supramolecular activation complex (pSMAC) that surrounds the TCR-peptide-MHC complexes localised at the centre of the SMAC in the IS [7]. We failed to detect VCAM-1 expression by DC in the spleen either prior to or during infection, thus questioning the potential for VLA-4/VCAM-1 interactions in the SMAC of any IS that formed between DC presenting parasite antigen and *L. donovani*-specific T cells in the spleen after infection. However, we cannot rule out the possibility that physiologically relevant VCAM-1 expression on splenic DC is present, but beyond the detection limits of the histological and FACS methods we have employed. All conventional DC subsets in the spleen are capable of T cell activation, and the CD8^+^ DC isolated from *L. donovani*-infected mice 5 hours p.i. promote IL-12/23p40-dependent skewing towards IFNγ production by responding CD4^+^ T cells [27]. In addition, the CD8^+^ DC are capable of acquiring *L. donovani* amastigotes independent of VCAM-1 early after infection, and these infected cells are able to produce IL-12p40 (Maroof, unpublished). Our data also indicate that VCAM-1/VLA-4 interactions play an important role in the priming of parasite-specific CD4^+^ T cells.

Short-term (5 hr) experiments with anti-VCAM-1 Fab′ fragments suggested that FcγR ligation on DC was not responsible for the observed reduced DC IL-12p40 production. However, blockade of VCAM-1 and the reduction of DC IL-12p40 with Fab′ fragments was not as effective as whole mAb, perhaps due to their extremely short half-life and rapid excretion by the kidney due to their low molecular weight [24],[25]. Ideally we would have liked to confirm our results at day 14 with Fab′ fragments, as well as whole mAb. However, it would not be possible to interpret the results of such long-term experiments accurately due to the limitations discussed above.

In conclusion, we have shown that VCAM-1/VLA-4 interactions modulate CD8^+^ DC IL-12p40 production and may play a role in the activation of parasite-specific CD4^+^ T cells during disease. Furthermore, our data indicate that VCAM-1 and VLA-4 are not directly involved in cellular recruitment to the liver during VL. These findings advance our understanding of the induction of cell-mediated immune responses following pathogen challenge, and identify a potential target for modulation to either enhance or suppress inflammation during disease.

## Materials and Methods

### Mice

Inbred female C57BL/6 and BALB/c mice were purchased from the Australian Resource Centre (Canning Vale, Western Australia), and maintained under conventional conditions. All mice used were age-matched (6 to 10 weeks), and were housed under specific-pathogen free conditions. All animal procedures were approved and monitored by the Queensland Institute of Medical Research Animal Ethics Committee.

### Parasites and infection of mice

*L. donovani* (LV9) was maintained by passage in BALB/c or B6.RAG-1^−/−^ mice, and amastigotes were isolated from the spleens of chronically infected mice. Mice were infected by injecting 2×10^7^ amastigotes intravenously via the lateral tail vein, killed at the times indicated in the text by CO_2_ asphyxiation and bled via cardiac puncture. In experiments examining DC IL-12p40 protein production, mice were infected with 1×10^8^ amastigotes intravenously, as previously reported [15],[18],[27]. Spleens and perfused livers were removed and parasite burdens were determined from Diff-Quick-stained impression smears (Lab Aids, Narrabeen, Australia), and expressed in Leishman-Donovan units (the number of amastigotes per host nuclei multiplied by the organ weight) [28]. Liver and spleen tissue were also preserved in either RNA*later* (Sigma-Aldrich, Castle Hill, Australia) or Tissue-Tek O.C.T. Compound (Sakura, Torrance, USA). Hepatic mononuclear cells (MNC) were isolated immediately following death as previously described [17].

### VCAM-1 and VLA-4 blockade

Anti-VCAM-1 (MK2/7; CRL-1909, rat IgG1) [29] and anti-VLA-4 (P/S2; CRL-1911, rat IgG2b) [30] hybridomas were purchased from the American Type Culture Collection (Manassas, VA). Purified antibody was prepared from culture supernatants by protein G column purification (Amersham, Uppsala, Sweden) followed by endotoxin removal (Mustang membranes, Pall, East Hills, NY). For VCAM-1 and VLA-4 blockade, C57BL/6 mice were injected i.p. with 1 mg of appropriate mAb or purified control rat IgG (Sigma-Aldrich) on the day of infection and every three days thereafter, or as detailed in the text. This dosing regime was based on one previously used in a collagen-induced arthritis model [31], except that 0.5 mg doses were used in this study. In our hands, 0.5 mg doses only achieved partial blockade of DC IL-12p40mRNA accumulation and hepatic anti-parasitic immunity, compared with 1 mg doses, hence our use of the increased amounts of mAb. We also found that the anti-VLA-4 mAb could be detected on the surface of splenic lymphocytes for at least 72 hours following injection of 1 mg into naïve mice (data not shown). Anti-VCAM-1 mAb and control rat IgG Fab fragments were generated using a commercial kit according to the manufacturer's instructions (Thermo Scientific, Rockford, IL).

### Histological response to hepatic infection

Acetone-fixed liver sections (6 µm) were labelled with hamster antisera to *L. donovani* amastigotes at a dilution of 1 in 1000. Labelling was detected with a biotinylated goat anti-hamster antibody (Vector Laboratories, Burlingame, CA). Sections were developed with Vector-Elite ABC kit, followed by 3,3′-diaminobenzidine substrate kit (Vector Laboratories). Granuloma density was determined from 25 fields of view per mouse liver (×40 magnification), and the maturation of granulomas was scored around infected Kupffer cells, as described elsewhere [32].

### Isolation of DC

Spleens were digested in collagenase type IV (1 mg/ml; Worthington, Lakewood, NJ) and deoxyribonuclease I (0.5 mg/ml; Worthington) at room temperature for 45 minutes. Splenocytes were isolated by passing digested spleens through a 100 µm cell strainer, followed by red blood cell lysis (Sigma-Aldrich). CD11c^+^ DC were positively selected from splenocyte preparations using magnetic-activated cell sorting (MACS) with metallo-conjugated anti-mouse CD11c antibodies (N418) and positive selection columns, according to the manufacturer's instructions (Miltenyi Biotec, Bergisch Gladbach, Germany). In some experiments, following MACS enrichment, DC were sorted into CD8α-positive and CD8α-negative populations by labelling with antibodies to CD11c, MHC-II and CD8α, and sorting on a MoFlo Cell Sorter (Dako, Botany, NSW, Australia), as shown in Figure 7.

### Flow cytometry

Liver MNC or splenocytes were harvested and pre-incubated with CD16/32 mAb (2.4G2; grown in-house) to avoid non-specific binding of antibodies to FcγR. For the staining of cell surface antigens, cells were incubated with fluorochrome-conjugated or biotinylated mAbs on ice for 30 minutes followed by streptavidin incubation for an additional 30 minutes when required. T cells, NKT cells and NK cells were enumerated with allophycocyanin (APC)-conjugated anti-TCRβ chain (H57-597), fluorescein isothiocyanate (FITC)-conjugated anti-CD4 (GK1.5), phycoerythrin (PE)-conjugated anti-CD8α (H1.2F3), and biotinylated anti-NK1.1 (PK136). B cells were enumerated using FITC-conjugated anti-CD19 (6D5) and APC-conjugated anti-B220 (RA3-6B2). DC were enumerated with APC-conjugated anti-CD11c (N418) and FITC- or PE-conjugated anti-I-A/I-E (MHC-II; M5/114.15.2). All mAbs were purchased from Biolegend (San Diego, CA) or BD Biosciences (Franklin Lakes, NJ). Rat anti-mouse CR3 (5C6) and rat anti-mouse GR-1 (RB6 8C5) were grown and biotinylated in house, and used to enumerate monocytes and granulocytes, respectively. Biotinylated antibodies were detected using Alexa Fluor 488-conjugated streptavidin (Invitrogen Life Technologies, Mount Waverley, Australia). Flow cytometric analysis was performed on a FACScalibur flow cytometer and analysed using Cell Quest Pro Software (BD Biosciences). For intracellular IL-12p40 staining, splenocytes were incubated for 4 hours at 37°C in 10% (v/v) foetal calf serum, RPMI containing 10 µg/ml brefeldin A (Sigma-Aldrich) prior to cell surface labelling with antibodies to CD11c, CD8α and CD4 (all from BD Biosciences). Cells were then washed and fixed in 1% (w/v) paraformaldehyde, before being washed in FACS buffer containing 0.1% (w/v) saponin (BDH, Lutterworth, UK) and stained with PE-conjugated anti-IL-12p40 (C15.6) or an isotype control mAb (both from BD Biosciences).

### DC cytospins

Sorted CD8^+^ and CD8^−^ DC (1×10^5^) in 100 µl FACS buffer were collected onto a glass slide using a Cytospin 3 centrifuge, according to the manufacturer's instructions (Shandon Scientific Ltd, Cheshire, UK), prior to staining with Diff-Quick (Lab Aids) to visualise host cell and parasite nuclei microscopically.

### Real Time Reverse Transcriptase-Polymerase Chain Reaction

Total RNA was extracted from bone marrow, spleen or liver tissue using TRIzol reagent (Invitrogen Life Technologies), and an RNeasy Mini Kit with on-column DNase digestion (Qiagen, Valencia, CA). Total RNA was extracted from purified DC using an RNeasy Mini Kit with on-column DNase digestion (Qiagen), according to the manufacturer's instructions. RNA samples were reverse transcribed into cDNA using the cDNA Archive Kit (Applied Biosystems, Foster City, CA) according to the manufacturer's instructions. The number of IFNγ, TNF and NOS-2 cDNA molecules in each sample were calculated using Taqman Gene Expression Assays (Applied Biosystems), and the number of IL-12p40 (5′ CTTGCAGATGAAGCCTTTGAAGA (forward) and 5′ GGAACGCACCTTTCTGGTTACA (reverse)), and HPRT (5′ GTTGGATACAGGCCAGACTTTGTTG (forward) and 5′GATTCAACCTTGCGCTCATCTTAGGC (reverse)) (house-keeping gene) cDNA molecules in each sample were calculated by real-time reverse transcriptase-polymerase chain reaction (rtPCR) using Platinum Sybr Green Master Mix (Invitrogen Life Technologies). All real-time reverse transcriptase-polymerase chain reactions (rtPCR) were performed on a Corbett Research RG-3000 Rotor Gene (Corbett Life Sciences, Sydney, Australia). Standard curves were generated with known amounts of cDNA for each gene, and the number of cytokine molecules per 1000 HPRT molecules in each sample was calculated.

### Lymphocyte and DC trafficking experiments

Mice were pre-injected with 100 µg FITC-dextran i.v. (200,000 MW, anionic, Invitrogen Life Technologies) to label marginal zone macrophages, followed by 1 mg of anti-VCAM-1 or rat IgG control antibody i.p. 24 hours later. Lymphocytes were isolated from naïve splenocytes using Histopaque 1083 (Sigma), according to the manufacturer's instructions. Splenic lymphocytes or CD11c^+^ DC were labelled with Hoechst 33342, as described previously [12]. Mice were administered with 1×10^7^ Hoechst 33342-labelled lymphocytes or 1×10^6^ Hoechst 33342-labelled DC via the lateral tail vein 1 hour post mAb injection. Mice were sacrificed 3 hours following lymphocyte transfer and 24 hr following DC transfer, and spleens were removed and embedded in Tissue-Tek O.C.T. compound (Sakura). The distribution of Hoechst 33342-labelled cells was analysed in 20 µm sections mounted in Pro-long Gold anti-fade (Invitrogen Life Technologies) using a Carl Zeiss inverted fluorescent microscope under UV illumination.

### Confocal microscopy

Tissue-Tek O.C.T. compound-preserved sections (6 µm) of spleen tissue were acetone fixed and labelled with anti-VCAM-1 (429; MVCAM.A, BD Bioscience) detected by direct conjugation to Alexa Fluor 647 using a monoclonal antibody labelling kit, or with a fluorochrome conjugated goat anti-rat antibody (both from Invitrogen Life Technologies). To identify cell populations the sections were then labelled with different combinations of rat antibodies to murine metallophilic macrophages (MOMA-1, Acris Antibodies, Hiddenhausen, Germany), marginal zone (MZ) macrophages (ERTR9, specific ICAM-3-grabbing nonintegrin-related 1 (SIGNR1), Bachem Ltd. Merseyside, UK), reticular fibroblasts (ERTR7, BMA Biomedicals, Augst, Switzerland), endothelial cells (Meca-32, BD Biosciences), FITC -conjugated CD11c (BD Biosciences) and Alexa Fluor 647-conjugated F4/80 (BD Biosciences). Fluorochrome conjugated goat anti-rat antibodies were used for detection of purified antibodies. Sections were mounted in Pro-long Gold anti-fade (Invitrogen Life Technologies) and visualized using a Carl Zeiss inverted LSM META 510 confocal microscope.

### Antigen re-stimulation of splenic CD4^+^ T cells

Splenic CD4^+^ T cells were positively selected by MACS from splenocytes using metallo-conjugated anti-CD4 antibodies and positive selection columns, according to the manufacturer's instructions (Miltenyi Biotec). CD4^+^ T cells (5×10^4^ cells per well) were stimulated with 2×10^6^ paraformaldehyde-fixed *L. donovani* amastigotes, and 1×10^6^ irradiated, naïve C57BL/6 spleen cells at 37°C, 5% (v/v) CO_2_. After 72 hours of culture, cells were pulsed with 1 µCi [^3^H] thymidine for 18 hours, prior to measuring thymidine incorporation using a Betaplate reader, (Wallac, Turku, Finland).

### Statistical analysis

The statistical significance of differences between groups was determined using a Mann Whitney test or an unpaired Student's *t* test using GraphPad Prism version 4.03 for Windows (GraphPad Software, San Diego, CA) and p<0.05 was considered statistically significant. The distribution of hepatic histological responses were compared using X^2^ analysis with Microsoft Excel software. All data are presented as the mean values±standard errors unless otherwise stated.
